# Spliceosomal introns in *Trichomonas vaginalis* revisited

**DOI:** 10.1186/s13071-018-3196-7

**Published:** 2018-11-27

**Authors:** Shuqi E. Wang, Abdul S. Amir, Tai Nguyen, Anthony M. Poole, Augusto Simoes-Barbosa

**Affiliations:** School of Biological Sciences, University of Auckland, Auckland, New Zealand

**Keywords:** *Trichomonas vaginalis*, Splicing, Spliceosome, Introns, Deep-branching eukaryote

## Abstract

**Background:**

The human protozoan parasite *Trichomonas vaginalis* is an organism of interest for understanding eukaryotic evolution. Despite having an unusually large genome and a rich gene repertoire among protists, spliceosomal introns in *T. vaginalis* appear rare: only 62 putative introns have been annotated in this genome, and little or no experimental evidence exists to back up these predictions.

**Results:**

This study revisited the 62 annotated introns of *T. vaginalis* derived from the genome sequencing plus previous publications. After experimental validation and a new genome-wide search, we confirmed the presence of introns in 32 genes and 18 others were concluded to be intronless. Sequence analyses classified the validated introns into two types, based on distinctive features such as length and conservation of splice site motifs.

**Conclusions:**

Our study provides an updated list of intron-containing genes in the genome of *T. vaginalis*. Our findings suggests the existence of two intron ‘families’ spread among *T. vaginalis* protein-coding genes. Additional studies are needed to understand the functional separation of these two classes of introns and to assess the existence of further introns in the *T. vaginalis* genome.

**Electronic supplementary material:**

The online version of this article (10.1186/s13071-018-3196-7) contains supplementary material, which is available to authorized users.

## Background

Introns, intervening non-coding sequences in genes of eukaryotes, are precisely removed from pre-mRNA by splicing, yielding mature mRNA. Splicing is achieved by a ribonucleoprotein complex (i.e. the spliceosome) through recognition of sequence elements on the introns such as the 5' and 3' splice sites (SS) and the branch site (BS) [1]. Phylogenomic analyses indicate that components of the spliceosome are conserved across eukaryotes [2], and that the last common ancestor of all eukaryotes contained an intron-rich genome [3, 4]. That said, identification of spliceosomal introns and small nuclear RNAs often requires careful experimental study [5–7].

One species where our understanding of introns remains particularly patchy is *Trichomonas vaginalis*. This is a protozoan parasite of the human urogenital tract and the causative agent of trichomoniasis, the most prevalent non-viral sexually transmitted infection worldwide [8]. As a member of the Excavata, a major eukaryotic supergroup [9, 10], *T. vaginalis* has experienced a recent genome expansion as a result of gene duplications, transposon activities and lateral gene transfer [11, 12]. The relatively large genome of *T. vaginalis* (~170 Mbp) harbours a protein-coding capacity of ~60,000 genes [11], and there is evidence of expression for about half of these [13, 14]. Despite this large gene repertoire, the number of *T. vaginalis* genes that contain spliceosomal introns is predicted to be very low (~0.001 introns/gene) [11, 15, 16]. Unicellular eukaryotes often have intron-poor genomes as compared to metazoans and plants but exceptions exist [17–21]. *Kipferlia bialata*, also a member of the Excavata, exhibits an average of ~7 introns/gene which is similar to the most intron-rich eukaryotic genomes [20].

While intron density appears low in *T. vaginalis* genome, it is not clear whether this is a result of natural variation or limited data. It is also worth noting that only a very few introns have been assigned to protein-coding genes of *T. vaginalis* experimentally [15, 16]. Vanacova et al. [15] were the first to demonstrate splicing activity in *T. vaginalis*. A 35-nt intron, modified from *Giardia lamblia* [22], was shown to be spliced out from a reporter gene in *T. vaginalis* [15]. Mutagenesis demonstrated a requirement for conserved splicing motifs and searching for this strict motif yielded the identification of 41 putative introns in *T. vaginalis* protein-coding genes [15]. However, searching for a strict motif, based solely on mutagenesis of a single *Giardia*-derived intron [15], suggests that other introns might exist. Indeed, Deng et al. [16] identified a 25nt-long intron in the gene *Rab1a* of *T. vaginalis* where the splicing motifs did not match to the strict motif used by the previous report [15]. The draft genome of *T. vaginalis*, released between the aforementioned studies, included the annotation of 62 introns among the ~60,000 predicted protein-coding genes [11].

Given the limited data currently available, it is therefore difficult to ascertain whether these observations indicate that *T. vaginalis* has an intron-poor genome and short introns with conserved SS and BS, or whether other introns may be present in the genome. As a first step towards addressing this question, we revisited all *T. vaginalis* introns that were predicted from the genome annotation and described in previous publications [11, 15, 16]. We applied reverse transcription and PCR to validate all introns in *T. vaginalis* experimentally. Splicing activity was confirmed by sequencing the exon-exon boundaries of the spliced products. Our results support the existence of two types of introns in *T. vaginalis* with distinctive length, SS and BS features.

## Methods

### *Trichomonas vaginalis* culture and purification of nucleic acids

*T. vaginalis* strain G3 was cultured in Diamond’s media [23] supplemented with 10% heat-inactivated horse serum, penicillin (1000 units/ml) and streptomycin (0.1 mg/ml). Genomic DNA (gDNA) was isolated using a modified protocol [24]. Briefly, a total of 10 ml of cells (10^6^ cells/ml) were washed and resuspended in 0.9 ml of phosphate-saline buffer. Cells were lysed by adding 0.1 ml of lysis buffer (8M urea; 2% sarkosyl; 150 mM NaCl, 1 mM EDTA and 100 mM Tris-HCl pH 7.5). Lysis was followed by phenol and chloroform extractions and the DNA/RNA from the aqueous phase was precipitated with 0.6 volumes of isopropanol. After washing with 70% ethanol and air-drying, the pellet was resuspended in 0.5 ml of TE buffer (10 mM Tris-HCl pH 8.0; 1 mM EDTA) followed by digestion with RNase A (5 μg/ml) and stored for use. Total RNA was extracted using TRIzol Reagent (Invitrogen, Waltham, USA) and treated with DNase I (New England Biolabs, Ipswich, USA) to remove gDNA contamination.

### Retrieval of multi-exon genes

Sequences of the 61 *T. vaginalis* protein-coding genes with exon count ≥ 2 were downloaded from trichdb.org (total 62 introns). The available features of the 42 intron-containing sequences described by the two early studies (i.e. full or partial sequences, intron length, 5' and 3' SS, flanking exon sequences) were extracted from the original publications [15, 16] and used to match against the genome annotation of *T. vaginalis* strain G3. The old gene IDs given by the early publications were replaced with the TrichDB ID if the intron fragment was located in a protein-coding gene according to the current genome annotation.

### Experimental validation of introns

Splicing activity was assessed by reverse transcription and PCR (RT-PCR). The first-strand, complementary DNA (cDNA) was synthesised from the purified total RNA using SuperScript III Reverse Transcriptase (RT) and oligo dT primer as recommended (Thermo Fisher Scientific, Waltham, USA). gDNA and cDNA were used as templates for polymerase chain reaction (PCR) using specific primers that target the flanking regions of the putative intronic sequences (Additional file 1: Table S1). As negative controls, water and a cDNA sample without reverse transcriptase (-RT) were used in the PCR reactions instead of gDNA or cDNA templates. PCR reactions were initially carried out using DreamTaq DNA Polymerase, as recommended (Thermo Fisher Scientific). Alternatively, for those PCR reactions that did not yield clear results, Phusion DNA Polymerase was used with either buffers as provided (Thermo Fisher Scientific) and by optimizing annealing temperature as detailed in Additional file 1: Table S1. Along with a 100 bp DNA ladder, PCR products from each set of primers were compared side by side by electrophoresis on a 2% (w/v) agarose in a Tris-Borate-EDTA buffer containing 0.5 μg/ml ethidium bromide (Thermo Fisher Scientific). For all newly discovered intron-containing genes in this study, the RT-PCR amplicons of the spliced products were eluted from the agarose gels and the DNA was sequenced. Each sequencing result was manually aligned to the original gene sequence to determine the exact exon-exon boundary.

### Using RNA sequencing data to evaluate splicing activity of undetermined introns

A strategy based on the available RNA sequencing (RNA-Seq) data was used to evaluate the splicing activity of 13 introns that could not be determined experimentally, as described above (i.e. undetermined introns). The two largest and most recent *T. vaginalis* RNA-Seq datasets (SRX2311573 and SRX2311572) were downloaded from the Sequence Read Archive at the National Center for Biotechnology Information (https://www.ncbi.nlm.nih.gov/sra). These datasets contain expressed sequences of *T. vaginalis* strains G3 and B7RC2, respectively. Over 180 and 160 million sequencing reads of 125 bp long from these 2 datasets were imported to Geneious 11.1.2 separately and mapped to the 13 genes carrying the undetermined introns. The genes TVAG_416520 and TVAG_337250, containing a functional and a non-functional intron, were chosen as positive and negative controls, respectively. The number of sequencing reads from the RNA-Seq data covering each nucleotide position was determined for each gene. A marked drop in sequencing depth on the intron regions as compared to their respective flanking exons, and in reference to controls above, was considered as evidence of splicing activity.

### Analyses of the intron features and the search for additional intron-containing genes

Sequence properties of the newly discovered introns in this study were described using a Python script which reads FASTA sequences one by one and retrieves all intron-related information as presented here. Introns were classified here into two different families, based on sequence characteristics and putative splicing motifs. The consensus sequences of the two intron families in *T. vaginalis* were updated, according to the previous studies on *T. vaginalis* introns [15, 16]. These two newly emerged patterns ('GYAYGYN{41,178}RCTAACACAYAG' and 'GTWYWDN{7}TCTAACH{1,2}AACAG', regular expressions correspond to Python syntax) were used to search *de novo* for matches on *T. vaginalis* genome segments using the DNA motif pattern search tool at TrichDB (http://trichdb.org/trichdb/showQuestion.do?questionFullName=SpanQuestions.DynSpansByMotifSearch). Potential hits found by this search were subjected to the same RT-PCR validation, as described above.

## Results

### An overview of the putative list of intron-containing genes in *T. vaginalis*

**Fig. 1:**
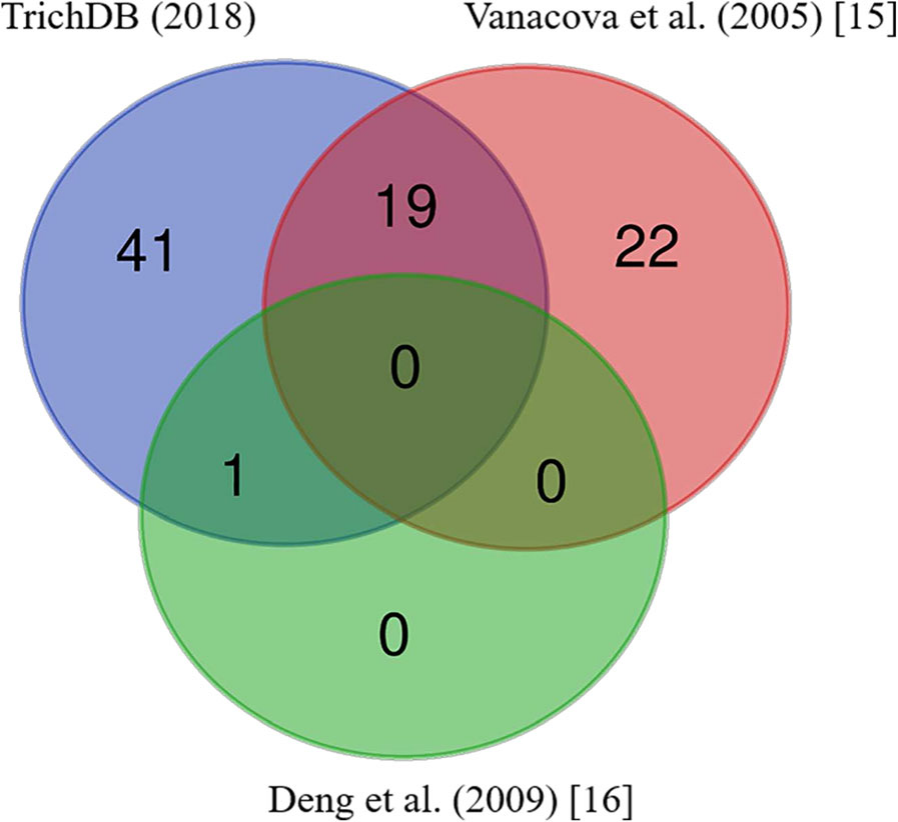
A Venn diagram representing the overlap on the number of introns that were predicted by TrichDB (blue) and reported by the previous publications (red: Vanacova et al. (2005) [15] and green: Deng et al. (2008) [16]) and as indicated

In this study, we interrogated two datasets of intron-containing genes in *T. vaginalis* (Fig. 1). The first dataset is represented by 61 genes, derived from the genome annotation. These genes contain 62 putative introns and are labelled as multi-exon genes by TrichDB (trichdb.org). The second dataset consists of 42 gene sequences, each carrying a single putative intron that were described in earlier publications [15, 16]. The available sequences of these 42 putative intron-containing genes, extracted from earlier publications [15, 16], were used to search for annotated genes in the *T. vaginalis* genome. Nineteen of the 41 putative intron fragments predicted by Vanacova et al. [15] were located in the transcribed region of protein-coding genes and annotated as multi-exon genes by TrichDB (Fig. 1). The remaining 22 fragments were exclusively present in non-transcribed intergenic regions (as supported by the EST data available at TrichDB), hence were not considered further. *TvRab1a*, the only intron-containing gene confirmed by Deng et al. [16], was annotated in TrichDB as one of the 61 multi-exon genes (Fig. 1). The strict motif sequence search used in the genome-wide search by Vanacova et al. [15] did not identify this intron (Fig. 1). The gene IDs were updated where applicable (Additional file 2: Table S2).

### Experimental validation of *T. vaginalis* introns

Primers flanking all 62 putative introns were designed to assess splicing activity using RT-PCR, gel electrophoresis and DNA sequencing (Additional file 1: Table S1). PCR reactions were performed on gDNA and cDNA templates. Negative PCR controls (water and -RT) were included to ensure that reactions were free of DNA contamination. While a single amplicon of expected size from the gDNA demonstrated the reliability of PCR, amplification from the cDNA demonstrates that the gene is transcribed. Size comparison between the amplicons produced from cDNA and gDNA should indicate if the intron has been spliced out. We also considered that both unspliced and spliced mRNAs can be represented in a cDNA sample, hence double bands may be observed.

Based on the expectations above, we classified introns into three mutually exclusive categories: (A) functional; (B) non-functional or (C) undetermined (Additional file 3: Figure S1). We identified 31 functional introns based on the detection of the spliced amplicon from the cDNA, always followed by unspliced amplicon from the gDNA. The cDNA may also produce the unspliced amplicon, indicated by double bands in some examples (Additional file 3: Figure S1a). Contrastingly, although the unspliced amplicon was detected from both the gDNA and cDNA, 18 introns were categorized as non-functional because the cDNA did not produce the spliced amplicon (Additional file 3: Figure S1b). The remaining 13 introns were classified as undetermined (Additional file 3: Figure S1c). This is because either the mRNA transcript was not detected (i.e. no amplicon from cDNA) or there were no size-reliable amplicons from cDNA and/or gDNA templates (such as a lack of amplification, smeary amplification or multiple bands), despite PCR optimization (as described in Methods and Additional file 1: Table S1).

To sum up, the experimental validation confirmed the presence of functional introns in all of the 20 protein-coding genes claimed by previous studies [15, 16] (Table 1, Fig. 1). Additionally, we were able to experimentally confirm the presence of 11 additional introns in protein-coding genes of *T. vaginalis*. Together, all 31 functional introns were found to be located in the coding sequence (CDS) of the protein-coding genes as one intron per gene. Finally, 18 introns in 17 protein-coding genes were assigned as non-functional as they were certainly transcribed but the predicted intronic sequences were not removed (Table 1).

**Table 1:** Summary of the PCR validation for the 62 putative introns distributed in 61 protein-coding genes as annotated by TrichDB

Category^a^	Gene ID^b^	Notes
(A) Functional introns	TVAG_110020, TVAG_390460, TVAG_126240, TVAG_225200, TVAG_087980, TVAG_413420, TVAG_388620, TVAG_176980, TVAG_053820, TVAG_020880, TVAG_110580, TVAG_350500, TVAG_148640, TVAG_460790, TVAG_198230, TVAG_125100, TVAG_085780, TVAG_065500, TVAG_014960	Described by Vanacova et al. [15]
	TVAG_383350	Described by Deng et al. [16]
	TVAG_324910, TVAG_416520, TVAG_134480, TVAG_089630, TVAG_043580, TVAG_306990, TVAG_147850, TVAG_217460, TVAG_242770, TVAG_056030, TVAG_203580	No early references
(B) Non-functional introns	TVAG_355610, TVAG_411060, TVAG_107710, TVAG_593670, TVAG_045310, **TVAG_130170**, TVAG_193820, TVAG_479870, **TVAG_410120**, TVAG_337250, TVAG_455320, TVAG_454570, **TVAG_288660**, TVAG_178900, TVAG_327510, TVAG_037940, TVAG_525530^c^	
(C) Undetermined introns	TVAG_066220, TVAG_115540, TVAG_249380, **TVAG_296070**, TVAG_347440, TVAG_442350, **TVAG_115550**, TVAG_264700, **TVAG_368250**, **TVAG_416890**, TVAG_478810, TVAG_432870, TVAG_458560	

^a^Based on the RT-PCR results (Additional file 3: Figure S1), these introns were categorized as (A) Functional, (B) Non-functional or (C) Undetermined

^b^Genes, where introns were predicted to be in the untranslated regions (UTRs) and not in the coding sequences (CDS), are shown in bold

^c^This is the only gene from the list that was claimed to contain 2 introns instead of 1

### Characterisation of the newly discovered intron-containing genes

**Fig. 2:**
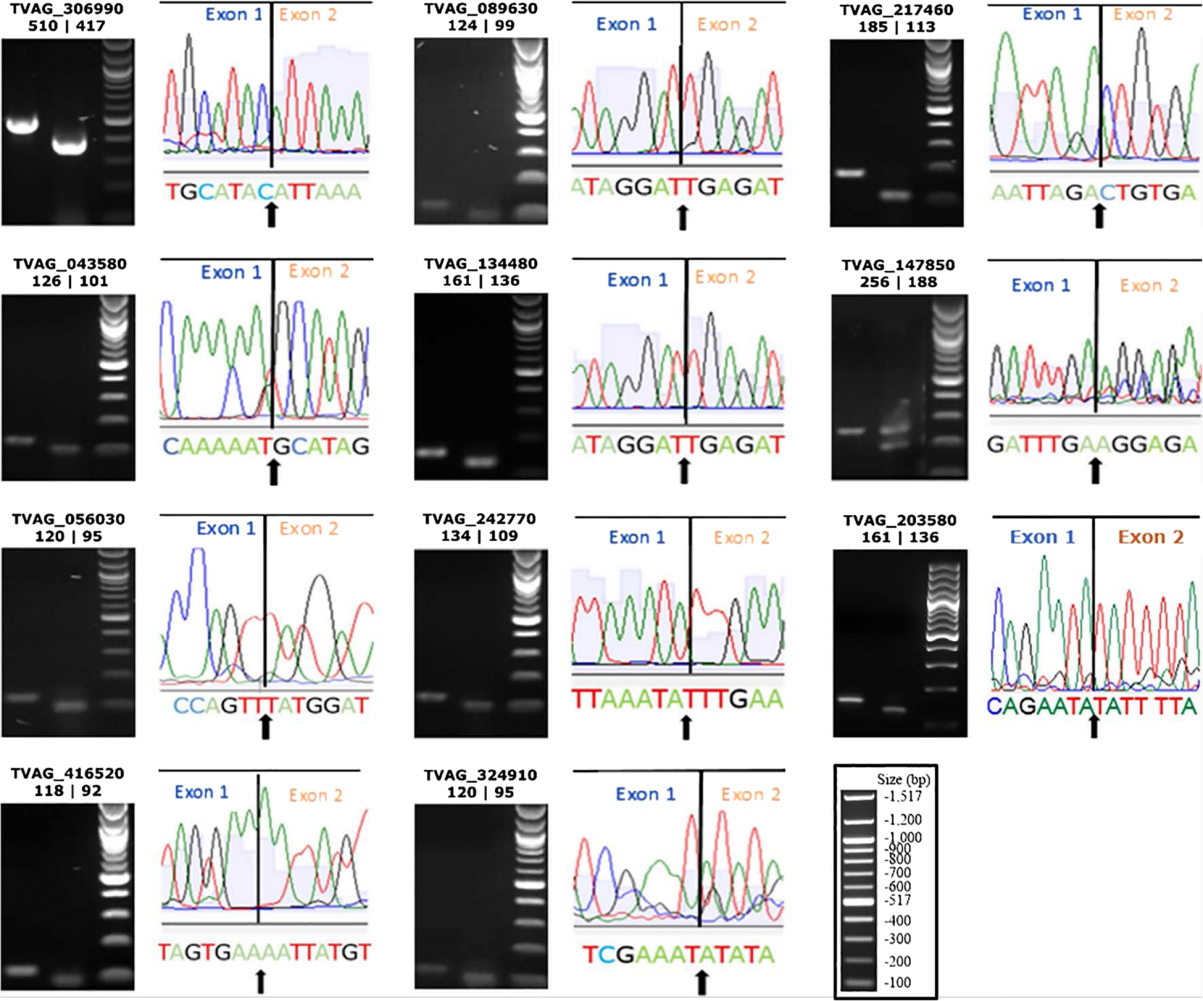
The exon boundaries of the 11 newly discovered intron-containing genes in *T. vaginalis*. Gel images, cropped from Additional file 3: Figure S1, show the unspliced and spliced amplicons in the first and second lanes, respectively followed by the 100 bp DNA ladder (New England Biolabs). The TrichDB ID of the genes is shown on the top of each gel image followed by the expected bp size of unspliced | spliced amplicons. Each gel image is accompanied by part of the DNA sequencing chromatogram where the line indicates the precise boundary between exon 1 and 2. The actual DNA sequence is shown under the chromatogram with the arrow indicating the nucleotide boundary between exons. The image in the box contains the 100 bp DNA ladder for reference

To confirm splicing activity of the 11 newly-discovered introns, the PCR products corresponding to the spliced amplicons were gel-purified and sequenced. DNA sequencing confirmed the predicted exon boundaries precisely for all 11 protein-coding genes interrupted by these introns (Fig. 2). The main features of these intron-containing genes are summarized in Table 2. They code for hypothetical proteins (6/11), kinases (4/11) and a eukaryotic protein belonging to the Mob1/phocein family (1/11). Eight of 11 introns were very short in length (25–26 nt), a feature shared with the short intron found in the *TvRab1a* gene [16]. Intronic sequences were shown to exhibit a lower GC content than exonic sequences, without exception (Table 2). Nine of 11 introns were in phases 1 or 2 (i.e. the intron interrupts a codon). Also, 9 of 11 introns were found in the first quarter of their open reading frames (Table 2).

**Table 2:** Features of the 11 newly discovered introns and intron-containing genes in *T. vaginalis*

Gene ID	Predicted function	Intron length (bp)	Exon/Intron GC content	IP^a^	RIP^b^	Intron phase	Exon/Exon nucleotide sequence	Exon/Exon amino acid sequence^c^
TVAG_306990	CMGC family protein kinase	93	43.18/29.03	307	0.25	0	ATAC/ATTA	LAY/IKA
TVAG_217460	Hypothetical protein	72	35.51/31.94	120	0.1	2	TAGA/CTGT	YEL/dCE
TVAG_147850	CAMK family protein kinase	68	36.72/25.0	533	0.45	1	CCAA/AATA	GSP/kYV
TVAG_416520	Hypothetical protein	26	41.63/30.77	109	0.17	0	TGAA/AATT	FSE/NYV
TVAG_043580	Mob1 phocein family	25	35.15/28.0	21	0.03	2	AAAT/GCAT	FSK/mHS
TVAG_056030	Hypothetical protein	25	40.33/24.0	137	0.42	1	GTTT/ATGG	RPV/yGL
TVAG_089630	AGC family protein kinase	25	37.39/24.0	78	0.06	2	GGAT/TGAG	DNR/iEI
TVAG_134480	Putative protein kinase	25	nd/24.0	78	0.08	2	GGAT/TGAG	DNR/iEI
TVAG_242770	Hypothetical protein	25	37.21/36.0	93	0.07	2	AATA/TTTG	IIK/yLK
TVAG_324910	Hypothetical protein	25	nd/24.0	327	nd	2	AAAT/ATAT	LIE/iYK
TVAG_203580	Hypothetical protein	25	34.77/24.0	165	0.12	2	AATA/TATT	LTE/yIL

^a^Intron position (IP) indicates the amino acid position of the intron relative to the first ATG in the open reading frame

^b^Relative intron position (RIP) indicates the intron position relative to the total gene ORF length

^c^Amino acids interrupted by phase 1 or 2 introns are shown in lower case

*Abbreviations*: nd, not determined (because of ambiguity of DNA sequence or incomplete length of CDS, as per TrichDB)

**Fig. 3:**
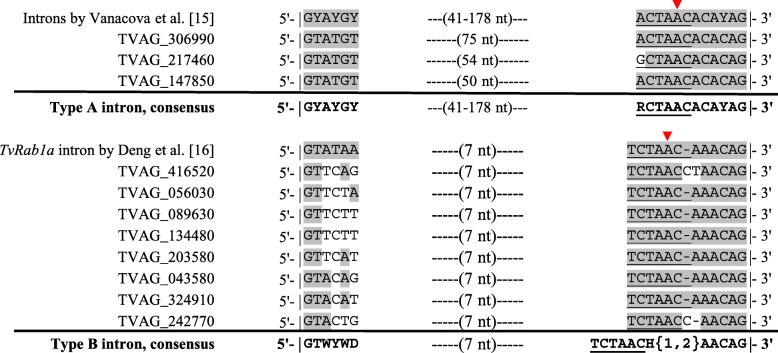
The 11 newly discovered *T. vaginalis* introns are classified into two types based on their sequence properties. Types A (top) and B (bottom) fit closely with the introns described by Vanacova et al. [15] and Deng et al. [16], respectively. The nucleotides of the newly discovered introns that are identical to the previously identified introns [15, 16] are shaded in grey. The branch site sequence, initially described as identical to the yeast consensus [26], is underlined with the red arrowhead indicating the branch adenosine. The distance in nucleotides (nt) between the 5' SS and the motif that encompasses the BS and 3' SS is indicated. Based on the intron nucleotide sequences, the consensus sequences for intron types A and B are shown below each alignment. Nucleotide ambiguity represents: ‘W’ to A or T; ‘Y’ to T or C; ‘H’ to A, C or T and ‘{1,2}’ specifies that ‘H’ can be one or two nucleotides

Manual inspection of the new 11 introns allowed us to classify them into two distinct types, named here types A and B, based on their sequence properties and splicing motifs (Fig. 3). Types A and B were represented by 3 and 8 introns and were closely related to the existing introns previously reported by Vanacova et al. [15] and Deng et al. [16], respectively. Type A introns show a one nucleotide mismatch to the strict consensus used for the genome-wide search from the previous study [15], precisely the first nucleotide on the 12nt-motif that encompasses the BS and the 3' SS. A guanosine instead of an adenosine at position one in this motif, just before the yeast consensus BS sequence ACTAAC [25], was found in the gene TVAG_217460. The intron length, however, was within the range of 41–178 nt as previously reported [15]. All three new introns of type A display the conserved 7-nt distance between the branch adenosine and the 3' SS, reiterating the previous observation [15] (Fig. 3).

Type B introns are notably short, between 25–26 nt long. In comparison to type A, they show a lower sequence conservation at the 5' SS. Also, the consensus BS sequence of type B introns places a T as the first nucleotide (TCTAAC) instead of A or G as in type A introns (RCTAAC). In contrast to type A introns, a space flexibility between the branch adenosine and the 3' SS is apparently allowed for type B introns. The type B intron found in the gene TVAG_416520 shows that this distance can be either 7 or 8 nt (Fig. 3).

### A genome-wide search for additional intron-containing genes

With a consensus sequences for intron types A and B (Fig. 3), we searched for additional intron-containing genes in the *T. vaginalis* genome. As a result, 52 and 32 genomic segments were found to contain the consensus sequences for introns types A and B, respectively (Additional file 4: Table S3, Additional file 5: Table S4). We identified 10 new sequences that resembled type A introns, but none were present in protein-coding genes (Additional file 4: Table S3). The other 22 segments, found in protein-coding genes, had already been validated by PCR in this study (Additional file 3: Figure S1, Additional file 4: Table S3). Therefore, no additional type A introns could be assigned to protein-coding genes from this new genome-wide search.

**Fig. 4:**
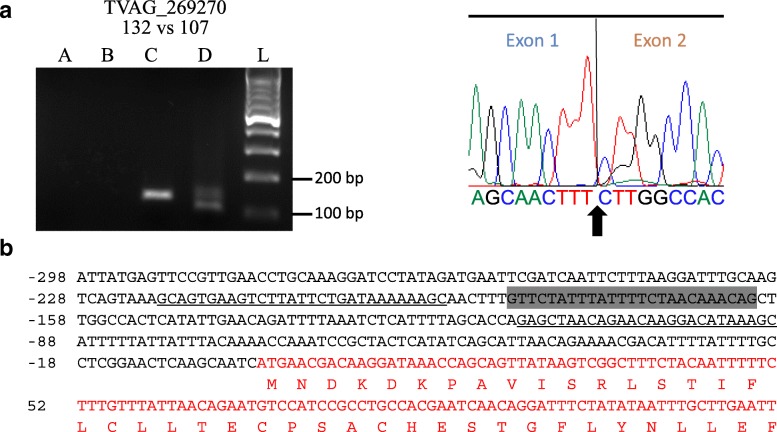
A newly identified type B intron in the gene TVAG_269270. a Experimental validation by RT-PCR and DNA sequencing. Left, the gel image of the RT-PCR is labelled with the gene ID on the top followed by the expected bp size of unspliced *vs* spliced amplicons. The lanes A-D were loaded with PCR products obtained from water, RNA, gDNA and cDNA templates, respectively. The lane L contains a molecular weight marker (100 bp DNA ladder by New England Biolabs) with band sizes indicated. Right, part of the DNA sequencing chromatogram where the line and arrow indicate the precise exon-exon boundary. The actual DNA sequence is shown under the chromatogram with the arrow indicating the nucleotide boundary between exons. **b** Partial sequence of gene TVAG_269270. The nucleotide sequence shows the entire 5' UTR where this 25nt-intron, highlighted in grey, was found. This is followed by part of the CDS sequence (in red) which is accompanied by its translation into one-letter amino acids. Primers surrounding the intron, which were used for the PCR here, are shown as underlined sequences. In contrast to the other characterized introns (Table 1), this type-B intron is found in the 5' UTR of the transcript

On the search for type B introns, however, a novel 25nt-long intron was found in a gene coding for a hypothetical protein (TVAG_269270). As this intron had not been experimentally validated (Additional file 3: Figure S1, Additional file 5: Table S4), we applied the same experimental approach to test the splicing activity of this intron (Fig. 4). PCR of gDNA and cDNA and sequencing of the spliced amplicon confirmed that this intron is spliced and enabled us to identify the exon-exon boundary (Fig. 4a). In contrast to all others, this intron is located within the 5' untranslated region (UTR) of the transcript (Fig. 4b).

## Discussion

This study confirmed the existence of 32 introns in protein-coding genes of *T. vaginalis*, as one intron per gene. Besides the 20 introns previously reported [15, 16], we validated another 12 introns in *T. vaginalis* experimentally. When revisiting introns in the *T. vaginalis* genome, we found that more than half of the putative intron segments reported previously [15] were not annotated in TrichDB. Similarly, we could not find experimental evidence for splicing activity for 31 putative introns in *T. vaginalis* multi-exon genes. More specifically, 18 of these could be classified as intronless genes (i.e. which do not appear to carry functional introns) since cDNA samples indicate expression, but only yielded full-length PCR products, consistent with the absence of splicing activity. We could not ascertain the splicing status for the remaining 13 putative introns experimentally. However, by mapping available *T. vaginalis* transcriptome reads to these genes *in silico*, we observed that all 13 genes are transcribed and that five of them show read mapping patterns consistent with functional introns (Additional file 6: Figure S2).

In examining the sequence properties of the 32 functional introns, confirmed here experimentally, these could be categorized into two types (named here A and B). Introns of type A conform to those previously reported by Vanacova et al. [15], except for the first nucleotide of the 12nt-motif that encompasses the BS and 3' SS. The change from ‘A’ to ‘G’ at this position was not considered in the original mutagenesis study [15]. Therefore, flexibility on this position may be allowed for splicing. Consistent with this, type B introns contain a ‘T’ at this position. Although the BS consensus for *T. vaginalis* does not seem as degenerate as for metazoans, it may be represented as a shorter consensus than initially observed [15], i.e. simply ‘CTAAC’ as seen in some Hemiacsomycete yeasts [26]. Despite re-considering this flexibility, no new introns of type A were found in protein-coding genes of *T. vaginalis* following our genome-wide search.

We confirmed the original observation of the positional conservation of the branch adenosine, precisely 7 nt away from the 3' SS [15], among type A introns. This feature of spliceosomal introns is shared between *T. vaginalis* [15, 16] and *G. lamblia* [27]. However, among type B introns, we identified one intron that challenged this positional conservation. Instead, the branch adenosine of the type B intron in TVAG_269270 was 8 nt away from the 3' SS. Mutagenesis studies are necessary to ascertain the exact proximity of the branch adenosine, relatively to the 3' SS of the pre-mRNA, that is necessary for splicing of type B introns in *T. vaginalis*.

In addition to the short length of type B introns, the low conservation of the 5' SS is another feature that separates them from type A introns according to the previous mutagenesis study [15]. Three of 10 type B introns contain the dinucleotide ‘AC’ in front of the canonical ‘GT’ at the 5' SS. This should prevent these introns from being spliced, according to the type A intron consensus and as experimentally supported [15]. However, based on our splicing activity assays, this is clearly not the case. In contrast to type A, a degenerate 5' SS seems to be allowed for type B introns. This feature was unexpected as a degenerate 5' SS is common to metazoans but not to yeast and protists [26]. Genomes of all protists that have experienced major intron losses had undergone strengthening of the 5' SS [21].

The type B intron found in TVAG_269270 after new genome search was the only one found in the UTR of a *T. vaginalis* transcript. All other introns were found in CDS of *T. vaginalis* protein-coding genes. The 5' UTRs of *T. vaginalis* mRNAs are known to be short in length, based on the close proximity between conserved core promoter elements and the ATG start codon [28]. These core promoter elements, which dictate transcription to initiate near the ATG start codon, are found in the large majority of *T. vaginalis* protein-coding genes [28]. The 5' UTR of this transcript, even after intron removal, does not seem to conform to this model. Although the distinctive features between intron types A and B described here suggest the existence of two intron families in *T. vaginalis*, further investigation is necessary to confirm the functional differences on sequence conservation of their splicing motifs.

In summary, we have examined evidence for splicing for a number of intron-containing genes in *T. vaginalis*. This study is by no means an exhaustive screen: however, it indicates that more introns might be present in this genome. As expected, standard intron-prediction models potentially fail when applied to genetically divergent eukaryotes such as *T. vaginalis*. Transcriptomic-based surveys, such as the one recently conducted for *G. lamblia* and *Spironucleus salmonicida* [27], will be useful to reveal the potential number of introns in the genome of *T. vaginalis*. These studies together should help close our gaps of understanding in the evolution of splicing, a signature of eukaryotic life.

## Conclusions

Our study provided an updated list of intron-containing genes in the *T. vaginalis* genome. A large number of misannotated introns indicates the inaccuracy of intron prediction algorithms used by genome projects when applied to a non-model eukaryotic organism. From this updated list, we were able to identify two potential intron families carrying distinctive features. A new intron consensus allowed us to discover one additional intron-containing gene, suggesting that further studies may expand the list of introns in the genome of this evolutionarily divergent eukaryote.

## Additional files

Additional file 1: **Table S1.** Primers used in this study to validate the putative introns by RT-PCR. Nucleotides that target 5' and 3' UTRs were indicated with lower-case letters. Underlined bases are located outside the transcribed region of these genes. (PDF 36 kb)

Additional file 2: **Table S2.** An update on the gene ID of the 42 introns previously reported, with references as indicated [15, 16]. (PDF 77 kb)

Additional file 3: **Figure S1.** Experimental validation of the 62 *T. vaginalis* putative introns by RT-PCR. These introns were categorised as (**a**) functional, (**b**) non-functional or (**c**) undetermined. (PDF 256 kb)

Additional file 4: **Table S3.** *T. vaginalis* genome segments that match the consensus sequence of type A intron (GYAYGYN{41,178}RCTAACACAYAG). (PDF 84 kb)

Additional file 5: **Table S4.** *T. vaginalis* genome segments that match the consensus sequence of type B intron (GTWYWDN{7}TCTAACH{1,2}AACAG). (PDF 85 kb)

Additional file 6: **Figure S2.** Evidence of gene transcription and mRNA splicing for the 13 putative introns that could not be experimentally determined in this study. (PDF 1422 kb)

## Abbreviations

BS: Branch site;
cDNA: Complementary DNA
CDS: Coding sequence
gDNA: Genomic DNA
IP: Intron position
RIP: Relative intron position
RNA-Seq: RNA Sequencing
RT: Reverse transcriptase
RT-PCR: Reverse transcription polymerase chain reaction
SS: Splice site
UTR: Untranslated region
