# Supplementary material for: Spliceosomal introns in Trichomonas vaginalis revisited
Source: Parasit Vectors. 2018 Nov 27;11:607. doi: 10.1186/s13071-018-3196-7 (PMC6260720; doi:10.1186/s13071-018-3196-7)
Supplement: Supplementary file 1 — Table S1. Primers used in this study to validate the putative introns by RT-PCR. Nucleotides that target 5' and 3' UTRs were indicated with lower-case letters. Underlined bases are located outside the transcribed region of these genes. (PDF 36 kb) [file 13071_2018_3196_MOESM1_ESM.pdf]

No.	Gene ID	Forward Primer Sequence (5' – 3')	Reverse Primer Sequence (5' – 3')	Intron Size (bp)	gDNA Product Length (bp)	cDNA product size (bp), if intron is spliced out	Notes
1	TVAG_014960	GGCAAGCATGTTTAAAAGGTGT	GGTGAAATTGCCGAGTCTTT	81	343	262	
2	TVAG_043580	tcagaaatttagtgcgATGAGC	AATCTTCATAGCACCGATGACC	25	126	101	a
3	TVAG_045310	GATACTGCGAAGTCAAGGAAGG	GCTCAACACGTGTGAAGAGTTC	104	691	587	b
4	TVAG_056030	CTGGAAAGAATATGGCAAGA	TCTGAGTAATCTGCGTGTCCAA	25	120	95	
5	TVAG_066220	TCAATCGAACAAGGTGTCACTC	ACATCTGGCTGGGTGTTAAAC	751	1574	823	b, h
6	TVAG_089630	GGTTGGCTCGAACGTAAGTT	TGTGCATTCTTCATCTTTCC	25	124	99	
7	TVAG_107710	CAACTTTCCAGTCATTGTTCCA	CTGATTTTGCTGTGTTTGCTGT	12	107	95	
8	TVAG_110580	CATACAGGGCTTCCCAAGAA	ACGCATATGCTCCCAGAATC	127	497	370	
9	TVAG_115540	ACTGGGATTCTCCATACTCACG	AGGCCACCAGTTTGTTTCTAAG	111	413	302	c, h
10	TVAG_126240	CTTCCTAATGGAAACCTTGACG	CATCGTTGGAGGGTAAATAGGA	99	527	428	
11	TVAG_130170	GCATGCAGTCCAAAAATTCA	gcctcgatcttagtgtctat	25	198	173	d, e
12	TVAG_134480	ATGTCAGGTTGGCTCGAACG	GCTGGGTAATTTCCATTTCAGA	25	161	136	
13	TVAG_193820	ATACCCTCCACAACCAGGTTAC	CATTCCTGGCTTACCCATTG	36	118	82	
14	TVAG_203580	AGCGTGGGGAGCTGAATTTGG	CTGCTATTCAATAGAATGTCAATGAA<br>CTG	25	161	136	h
15	TVAG_217460	CAGAAGGAGTTGAATGGAAATG	GCGACATGCATCAAAGTTAATC	72	185	113	
16	TVAG_242770	GGCGTGAGAACATTAAATGAAG	AAGCGTGGGATCCTATAACAGA	25	134	109	
17	TVAG_249380	AAGAAGAAAACCAGCGCAAA	GCGCTTAGCTTCTTCCTCAG	804	1177	373	h
18	TVAG_296070	gaagttgccgaactcaagaa	gctccttaagcttgtcttcg	51	157	106	c, d, h
19	TVAG_306990	GTATCCTTGCCAAATCCTTCTG	ACAGCTTCCGAAATCAACTAGC	93	510	417	
20	TVAG_324910	TCTTTCAGGCAGAAACCTCAGA	TATCAGCTCTTTGCTTTGCT	25	120	95	
21	TVAG_347440	TCCAACAGGCCCAATTACTATC	GTAAGCACCCTGTGGTGGAT	36	142	106	c, h
22	TVAG_350500	AGTTATTCGCGGTCAGCAGT	CGGTGGATGAGGTAATGGAC	114	423	309	
23	TVAG_383350	atccATGACTGCTAATGA	CAGAGAACATATCTTCGGCAAA	25	139	114	a
24	TVAG_416520	CTGCTTCAGTTTTCCGATCA	TGTCCGTCGATATCAAGAGTTC	26	118	92	
25	TVAG_442350	AAGGACTATCCGGAAGTGATGA	TGAGGCCAACTTTAGATCCTTT	64	168	104	h
26	TVAG_454570	GATGAAATCTTCGTTGCTCCAT	GGTGTAGTGCTGAGCTCCTTTC	291	827	536	
27	TVAG_455320	ACTTGGAATCGGTGGAAGAG	CGATCATCTGGGAGTTGACT	18	108	90	
28	TVAG_037940	TCTCCATCAAGTCAAACTTCTG	TGTTGATGGTTTTCCAAGATTTAAGC	66	255	189	
29	TVAG_085780	TTCTCTGCTTTTGAGTTCGATG	GGTTACCAACACTGCCTGAAAT	67	413	346	g
30	TVAG_115550	aggccaccagtttgtttctaag	accaaactggggttctccttat	112	307	195	d, h
31	TVAG_125100	CGATAAAGTAGGCGAAGGTCAT	GGGAGAGTCCTTCAAAATTCCT	105	407	302	g, h
32	TVAG_148640	TTACTTGCAGACTTCGGTTTTG	CTCGTCAGAGACATCGTCAAAG	93	354	261	
33	TVAG_176980	TCACAGCAGCATTATCAAGA	CCTGACAAGACTTATTGAAAGACC	59	278	219	
34	TVAG_178900	GAGCAGATTCATTCATACCAAG	ccttgagcatCTACTGTATGTT	43	172	129	a
35	TVAG_198230	GCAGGAATGCGTCATACAAC	CTGCCACCATGAGTTAGTCCTT	84	200	116	
36	TVAG_225200	CAGTGCTCCACCACAACCTA	TACATCGAGCACAAGGGTAA	76	248	172	
37	TVAG_264700	GTGGTATGCAGATTTTCGTCAA	CCTTTTGGATGGAGTAGTCCTG	228	423	195	h
38	TVAG_288660	TGAGTCCATctaacatcgaa	cagtgtggagctcatctgat	25	128	103	d, e
39	TVAG_337250	TAACCAGGGCTTTGGACAAC	ATCCTTGGTTAGCTGGCTGT	51	158	107	g
40	TVAG_355610	CAGAATCAGAACCAATTCAACG	GAAGCAGGTGGAGGATTAGAGA	40	179	139	
41	TVAG_368250	ATCAAGCATGTTAATGCTGGCA	ATGTCATAAaggcgtaagtg	35	169	134	d, e
42	TVAG_388620	GGTTCTTACCGTTTAGGTGTCG	TCGGCAAAATCTGTATCTTCTG	94	351	257	
43	TVAG_410120	tccatttgcacccctgaaat	gtgcgttagcattcaaaaac	25	201	177	d
44	TVAG_411060	CAAAGCACAAGAGAGAAGTTGC	GTTTGGGTTTTCACAGGACAAT	529	915	386	
45	TVAG_413420	TGATAGCTCTACGCAAGAAGCA	AACCGTAATACGTGGCTGTGTA	68	160	92	
46	TVAG_416890	ATCGAGATGTGACAGTAGTC	caacactacatttccagaatgc	1307	1740	433	d
47	TVAG_478810	GGCTAAAGAAGCTGCTGAAAAG	GCGTTTCTTGATTTCTTCTGCT	489	1128	639	
48	TVAG_479870	CAAGACCAAGACTTTCCAATGA	tctgagtactttgccatacc	40	199	159	a
49	TVAG_020880	CAAAGAAGCCCAAGAACTGACT	GAGGAGGACATTGTCTGGTTTC	196	686	490	
50	TVAG_053820	GATAGCTGACTTCGGTTTTGCT	CGTTGTGGTCTAACTCTTCACG	110	541	431	g
51	TVAG_065500	ACAGAATCAAGATTGCCGACTT	AAGATGGGTGTATCGGTGTAGG	134	536	402	
52	TVAG_087980	CCAGAGAAACATCGTGGTGATA	CGTCAGGAGGATCATAAAATGG	78	193	115	
53	TVAG_110020	AATCCATTTCTCGGCTACAGAA	CGGTGGTGCGTAGATATTGTTA	91	355	264	
54	TVAG_147850	GGCAGGAAAATACGATGAAAAC	CGAAAGAAAGCATTGGAAGAGT	68	256	188	
55	TVAG_327510	GGTAAATGGAGTCCAGCAGAGA	GATAACGCTGCTGTCAAGAATG	174	592	418	
56	TVAG_390460	CGACACGTTCGTTACCATATTC	AATCTGCGTGAGGATCAGAAAC	70	229	159	g
57	TVAG_432870	AAACAGAGCAGCCTGAACAG	TACCTGGTTGTTCTGGCT	2297	2422	125	h
58	TVAG_458560	CTTGTAGCTTCAGCTGGTATCG	GCAAGAGGGTAGAAATGTTTGG	1048	1507	459	h
59	TVAG_460790	TCTTCGTCATCCTCATGTTGTC	TTACTTCCACCACCAAAAGA	67	180	113	g
60	TVAG_525530	ACAGTTCAAAACACTGCACCAC<br>AACAAGTCCATCCTTTGGAAAC	TATCTGAACTTCTTCCTGTGGA<br>CCCCATCTCATTGTAGTTCC	405<br>290	552<br>427	147<br>137	f
61	TVAG_593670	TAACCAGCCTACTGAGCAGA	agttTTACTCATGTTGAATG	16	108	92	e

Notes: [a]One of the primers contains nucleotides from the UTR (lower-case); [b]The putative intron region contains undetermined nucleotide 'N'; [c]Alternative splicing was predicted on the gene; [d]The entire putative intron is located in UTR; [e]One of the primers includes nucleotides outside the mRNA (underlined); [f]The gene may contain 2 introns, hence 2 sets of primers were designed; [g]No amplicons were found under the normal PCR conditions but after optimisation (annealing temperature, polymerase and/or buffer) the target fragment was amplified; [h]no amplification or only non-specific amplicons were produced even after PCR optimisation (as described in methods).
