# Supplementary material for: Spliceosomal introns in Trichomonas vaginalis revisited
Source: Parasit Vectors. 2018 Nov 27;11:607. doi: 10.1186/s13071-018-3196-7 (PMC6260720; doi:10.1186/s13071-018-3196-7)
Supplement: Supplementary file 2 — Table S2. An update on the gene ID of the 42 introns previously reported, with references as indicated [15, 16]. (PDF 77 kb) [file 13071_2018_3196_MOESM2_ESM.pdf]

No.	Original Gene Name	Current Gene ID at TrichDB	Reference
1	PAP (94)	TVAG_388620	Vanacova *et al*. (2005) [15]
2	Cent (116)	None	
3	Cent (122)	None	
4	Cent (127)	TVAG_110580	
5	Scp (67)	TVAG_460790	
6	Scp (70)	TVAG_390460	
7	Scp (76)	TVAG_225200	
8	Taf6 (81)	TVAG_014960*	
9	Taf6 (91)	TVAG_110020	
10	STK (93)	TVAG_148640	
11	STK (70)	None	
12	STK (78)	TVAG_087980	
13	STK (59)	TVAG_176980	
14	STK (68)	TVAG_413420	
15	STK (105)	TVAG_125100	
16	STK (99)	TVAG_126240	
17	STK (110)	TVAG_053820	
18	STK (114)	TVAG_350500	
19	STK (134)	TVAG_065500	
20	STK (196)	TVAG_020880	
21	908787	None	
22	924721	None	
23	920443	TVAG_085780	
24	918654	None	
25	919336	None	
26	923879	TVAG_198230	
27	922157	None	
28	899749	None	
29	910641	None	
30	922300	None	
31	924085	None	
32	891036	None	
33	920747	None	
34	845470	None	
35	921979	None	
36	858605	None	
37	924111	None	
38	918865	None	
39	923921	None	
40	924029	None	
41	921720	None	
42	TvRab1a	TVAG_383350	Deng *et al*. (2008) [16]

**The nucleotides of the flanking exons of Taf6(81) should be 'tagc/agaa' as per TrichDB instead of 'tagc/aaca' as reported in the original reference [15].*
