# Supplementary material for: Spliceosomal introns in Trichomonas vaginalis revisited
Source: Parasit Vectors. 2018 Nov 27;11:607. doi: 10.1186/s13071-018-3196-7 (PMC6260720; doi:10.1186/s13071-018-3196-7)
Supplement: Supplementary file 3 — Figure S1. Experimental validation of the 62 T. vaginalis putative introns by RT-PCR. These introns were categorised as (a) functional, (b) non-functional or (c) undetermined. (PDF 256 kb) [file 13071_2018_3196_MOESM3_ESM.pdf]

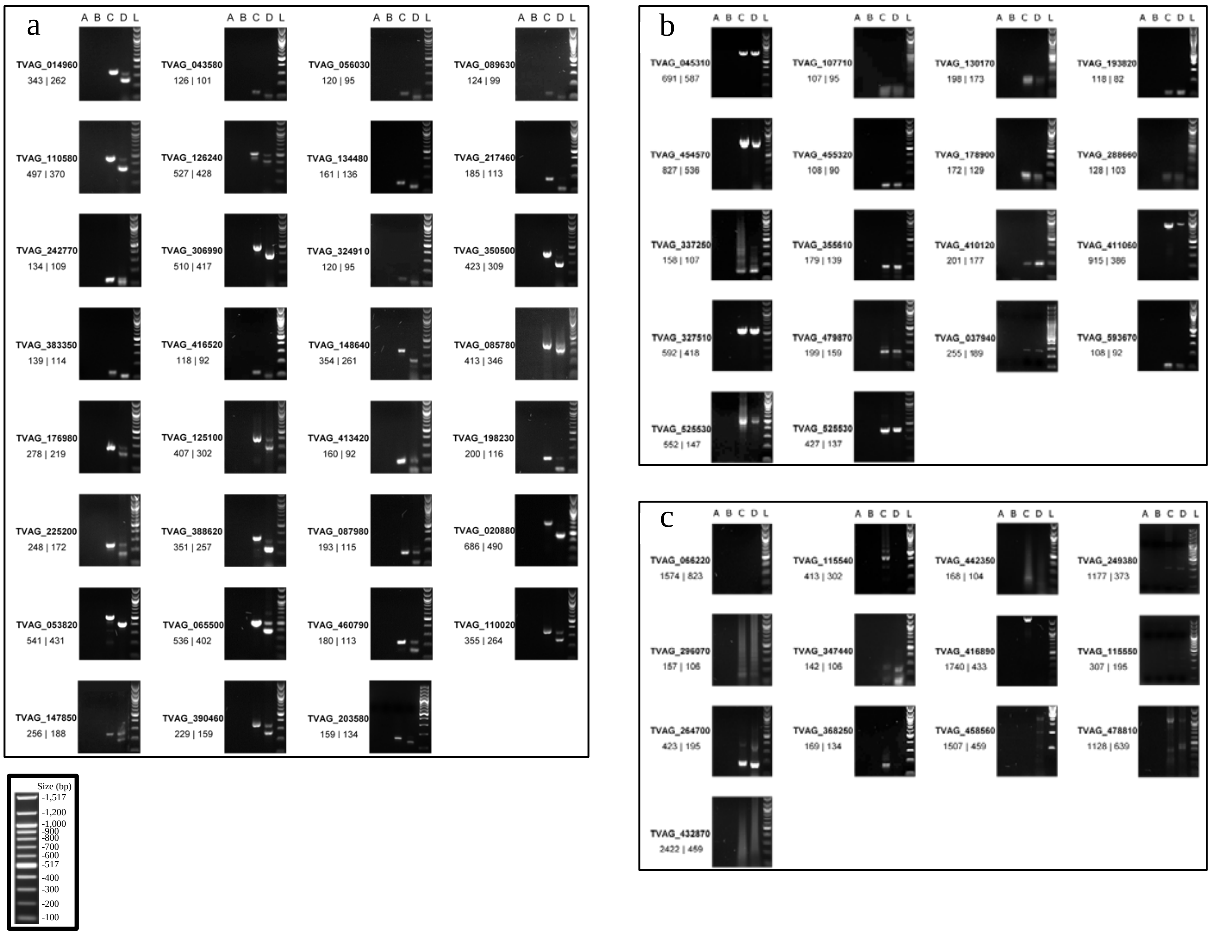

Notes: The lanes A-D were loaded with PCR products obtained from water, RNA, gDNA and cDNA templates, respectively. The lane L contains a molecular weight marker (100 bp DNA ladder by New England Biolabs) with band sizes indicated in the separate image. The gene ID is shown on the left side of each gel image followed by the expected bp size of unspliced | spliced amplicons.
