# Supplementary material for: Spliceosomal introns in Trichomonas vaginalis revisited
Source: Parasit Vectors. 2018 Nov 27;11:607. doi: 10.1186/s13071-018-3196-7 (PMC6260720; doi:10.1186/s13071-018-3196-7)
Supplement: Supplementary file 4 — Table S3. T. vaginalis genome segments that match the consensus sequence of type A intron (GYAYGYN{41,178}RCTAACACAYAG). (PDF 84 kb) [file 13071_2018_3196_MOESM4_ESM.pdf]

| Genomic Segment ID | Previous ID by Vanacova et al [15] [a] | Protein-coding gene ID, if annotated by TrichDB [b] | Intron validated in Fig S1? [c] |
|---|---|---|---|
| DS113189:290662-290745:r | 919336 | Not annotated | No |
| DS113190:59406-59490:f | 923879 | TVAG_198230 | Yes |
| DS113198:189344-189471:f | Cent (127) | TVAG_110580 | Yes |
| DS113198:45986-46077:f | Taf6 (91) | TVAG_110020 | Yes |
| DS113198:72036-72138:r | 922300 | Not annotated | No |
| DS113200:12132-12328:r | STK (196) | TVAG_020880 | Yes |
| DS113209:193807-193920:r | 858605 | Not annotated | No |
| DS113216:206121-206256:r | 899749 | Not annotated | No |
| DS113218:51116-51218:f | None | Not annotated | No |
| DS113224:8624-8700:r | Scp (76) | TVAG_225200 | Yes |
| DS113249:133470-133533:r | 924721 | Not annotated | No |
| DS113267:78748-78815:r | Scp (67) | TVAG_460790 | Yes |
| DS113269:100642-100736:f | PAP (94) | TVAG_388620 | Yes |
| DS113276:160463-160563:f | None | Not annotated | No |
| DS113279:161942-162045:f | None | Not annotated | No |
| DS113292:26357-26461:r | 924085 | Not annotated | No |
| DS113329:77767-77886:r | 924111 | Not annotated | No |
| DS113356:92090-92150:r | 908787 | Not annotated | No |
| DS113357:56818-56917:r | STK (99) | TVAG_126240 | Yes |
| DS113364:87506-87622:f | Cent (116) | Not annotated | No |
| DS113375:81424-81555:f | 924029 | Not annotated | No |
| DS113398:99395-99500:f | STK (105) | TVAG_125100 | Yes |
| DS113431:27837-27952:f | 922157 | Not annotated | No |
| DS113472:4998-5105:f | None | Not annotated | No |
| DS113477:61397-61511:r | STK (114) | TVAG_350500 | Yes |
| DS113480:48744-48814:r | Scp (70) | TVAG_390460 | Yes |
| DS113550:72375-72447:r | None | TVAG_217460 | Yes |
| DS113561:60331-60444:f | None | Not annotated | No |
| DS113572:54036-54147:r | 921979 | Not annotated | No |
| DS113577:26893-27023:r | 923921 | Not annotated | No |
| DS113607:10491-10620:r | 918865 | Not annotated | No |
| DS113624:21306-21384:r | STK (78) | TVAG_087980 | Yes |
| DS113672:49592-49662:r | STK (70) | Not annotated | No |
| DS113675:24387-24455:r | STK (68) | TVAG_413420 | Yes |
| DS113680:30508-30674:f | STK (59) | TVAG_176980 | Yes |
| DS113755:29856-29949:f | STK (93) | TVAG_148640 | Yes |
| DS113774:40351-40476:f | Taf6 (81) | TVAG_014960 | Yes |
| DS113785:34279-34389:f | STK (110) | TVAG_053820 | Yes |
| DS113789:42043-42143:r | 910641 | Not annotated | No |
| DS113792:26028-26111:f | 918654 | Not annotated | No |
| DS113880:26278-26368:r | None | Not annotated | No |
| DS113888:20779-20901:r | Cent (122) | Not annotated | No |
| DS113891:23458-23590:r | 921720 | Not annotated | No |
| DS114021:9497-9590:r | None | TVAG_306990 | Yes |
| DS114056:23850-23918:r | None | TVAG_147850 | Yes |
| DS114155:8502-8612:f | 845470 | Not annotated | No |
| DS114355:14790-14894:r | 891036 | Not annotated | No |
| DS114409:6762-6866:f | None | Not annotated | No |
| DS114439:11009-11076:f | 920443 | TVAG_085780 | Yes |
| DS114995:3256-3363:r | 920747 | Not annotated | No |
| DS115094:4588-4722:r | STK (134) | TVAG_065500 | Yes |
| DS120697:570-672:r | 922300 | Not annotated | No |

Notes: [a]The old segments, previously described [15], are shown with their original gene ID where applicable in this column. [b]Segments that are located in protein-coding genes were noted with the TrichDB ID where applicable in this column. [c]This column indicates if introns were experimentally validated as per Additional file 3: Figure S1.
