# Supplementary material for: Spliceosomal introns in Trichomonas vaginalis revisited
Source: Parasit Vectors. 2018 Nov 27;11:607. doi: 10.1186/s13071-018-3196-7 (PMC6260720; doi:10.1186/s13071-018-3196-7)
Supplement: Supplementary file 5 — Table S4. T. vaginalis genome segments that match the consensus sequence of type B intron (GTWYWDN{7}TCTAACH{1,2}AACAG). (PDF 85 kb) [file 13071_2018_3196_MOESM5_ESM.pdf]

Genomic Segment ID	Previous ID by Deng et al [16]	Protein-coding gene ID, if annotated by TrichDB [b]	Intron validated in Fig S1? [c]
DS113190:335792-335817:r	None	Not annotated	No
DS113212:132822-132847:r	None	Not annotated	No
DS113215:39171-39196:r	None	Not annotated	No
DS113232:135446-135471:r	None	Not annotated	No
DS113276:50161-50186:r	None	Not annotated	No
DS113305:53361-53386:f	None	Not annotated	No
DS113379:86879-86904:r [d]	None	TVAG_269270	No
DS113384:61202-61227:r	None	Not annotated	No
DS113419:42845-42870:f	None	TVAG_056030	Yes
DS113459:19860-19886:f	None	TVAG_416520	Yes
DS113486:15534-15559:r	None	TVAG_203580	Yes
DS113505:34256-34281:r	None	TVAG_043580	Yes
DS113513:82213-82238:r	None	Not annotated	No
DS113569:18673-18698:r	None	TVAG_324910	Yes
DS113640:40185-40210:f	None	Not annotated	No
DS113657:12549-12574:f	None	TVAG_242770	Yes
DS113657:41847-41872:r	None	Not annotated	No
DS113731:31075-31100:f	None	Not annotated	No
DS113785:16501-16526:f	None	Not annotated	No
DS113831:11705-11730:f	None	Not annotated	No
DS113839:34987-35012:f	None	Not annotated	No
DS113956:18734-18759:f	None	Not annotated	No
DS113985:30492-30517:f	TvRab1a [a]	TVAG_383350	Yes
DS114086:5861-5886:f	None	TVAG_134480	Yes
DS114221:16970-16995:r	None	TVAG_089630	Yes
DS114306:8464-8489:r	None	Not annotated	No
DS114386:14533-14558:f	None	Not annotated	No
DS114386:16239-16264:f	None	Not annotated	No
DS115898:3348-3373:r	None	Not annotated	No
DS117022:2384-2409:f	None	Not annotated	No
DS117874:2072-2097:r	None	Not annotated	No
DS129582:756-781:f	None	Not annotated	No

Notes: [a]The only old segment, previously described [16], is shown with its original name 'TvRab1a'. [b]Segments that are located in protein-coding genes were noted with the TrichDB ID where applicable in this column. [c]This column indicates if introns were experimentally validated as per Additional file 3: Figure S1. [d]This segment (in red) was found in a protein-coding gene (TVAG_269270, in red) containing an intron B consensus sequence that was not validated as per Additional file 3: Figure S1.
