# Supplementary material for: Spliceosomal introns in Trichomonas vaginalis revisited
Source: Parasit Vectors. 2018 Nov 27;11:607. doi: 10.1186/s13071-018-3196-7 (PMC6260720; doi:10.1186/s13071-018-3196-7)
Supplement: Supplementary file 6 — Figure S2. Evidence of gene transcription and mRNA splicing for the 13 putative introns that could not be experimentally determined in this study. (PDF 1422 kb) [file 13071_2018_3196_MOESM6_ESM.pdf]

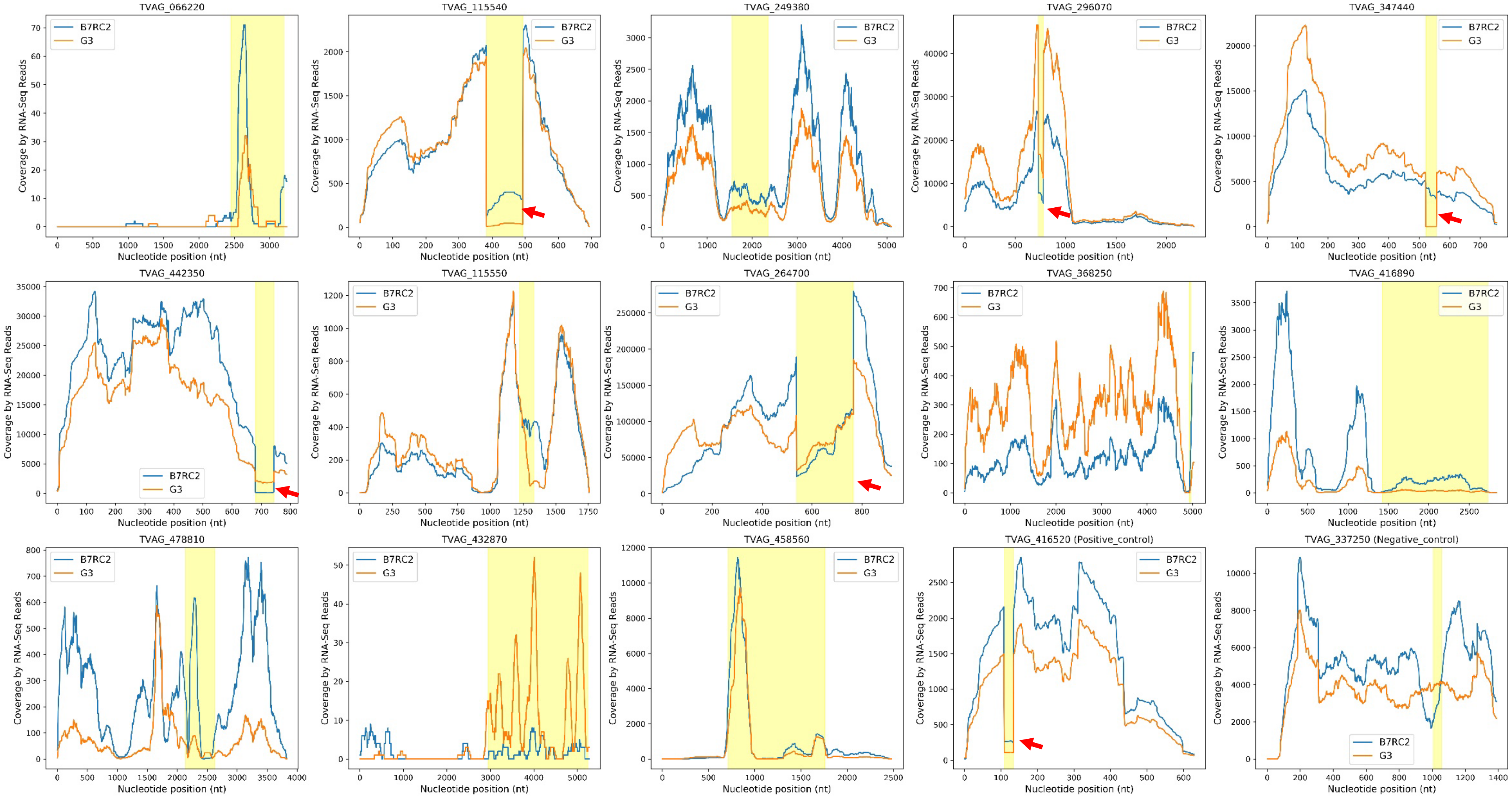

Notes: Details about these undetermined introns can be found in Table 1 and Additional file 3: Figure S1, under category C. Reads from RNA-Seq data for *T. vaginalis* strains G3 (orange line) and B7RC2 (blue line) were mapped back to the full sequence of these 13 genes. A functional (TVAG_416520) and a non-functional (TVAG_337250) intron were used as positive and negative controls. The number of sequencing reads covering each nucleotide position was counted as the depth. The predicted introns are shaded in yellow across the length of each gene. Five of these 13 putative introns show an abrupt reduction in read depth, similar to the positive control (indicated by the red arrow). This was detected for both G3 and B7RC2 strains, except for TVAG 347440 which was seen only for the genome strain G3. This significant drop of read depth was considered here as evidence of mRNA splicing, suggesting that these introns may be functional.
